# Modification of the fatty acid composition of the erythrocyte membrane in patients with chronic respiratory diseases

**DOI:** 10.1186/1476-511X-12-117

**Published:** 2013-07-30

**Authors:** Tatyana P Novgorodtseva, Yulia K Denisenko, Natalia V Zhukova, Marina V Antonyuk, Vera V Knyshova, Tatyana A Gvozdenko

**Affiliations:** 1 Vladivostok Branch of the Far Eastern Center of Physiology and Pathology of Respiration of SB RAMN, Institute of Medical Climatology and Rehabilitative Treatment, Vladivostok, Russia; 2 A.V. Zhirmunsky Institute of Marine Biology of the Far Eastern Branch of the Russian Academy of Sciences, Vladivostok, Russia; 3 Far Eastern Federal University, Vladivostok, Russia

**Keywords:** Chronic respiratory diseases, Fatty acids, Red blood cell membrane

## Abstract

**Background:**

Early preclinical diagnosis of COPD is urgent. We proposed that fatty acid composition of red blood cells may serve as a prognostic test for the complications in the chronic respiratory diseases. Fatty acid composition of the erythrocyte membranes in patients with chronic respiratory diseases (chronic bronchitis, CB, and stable chronic obstructive pulmonary disease, COPD) was studied. It was established that modification of the fatty acid composition in the erythrocyte membranes was unidirectional in both groups of patients.

**Methods:**

Patients with CB and stable COPD (group A, GOLD 1) (15 subjects in each group) were studied in clinic. The activity of the inflammatory process was evaluated by the phagocytic activity of neutrophils, cytokine levels and cytokine receptors in the blood serum (TNFα, sTNF-RI, bFGF, TGF-β, IL-8). Fatty acid (FA) composition of the erythrocyte membranes was analyzed by gas liquid chromatography. Statistical data processing was performed by the methods of descriptive statistics with Statistica 6.0.

**Results:**

In both groups (CB and COPD), a significant accumulation of the saturated FAs (14:0, 15:0, 18:0) was established. The amount of the arachidonic acid (20:4n-6) was increased by 13% (р < 0.05) in CB patients and by 41% (р < 0.001) in COPD patients, as compared with healthy persons. The elevated level of the PUFA n-6 in the erythrocytes membranes in patients with chronic respiratory diseases confirms that proinflammatory (leukotriene B4) and bronchospasm (prostaglandin D2) mediator substrates is increased. The level of the eicosapentaenoic acid (20:5n-3) was decreased by 32% (р < 0.05) in CB patients and 2-fold (р < 0.001) in COPD patients. The observed increase in the 20:4n-6/20:5n-3 ratio - 1.5-fold (р < 0.001) in CB patients and 3-fold in COPD patients - can be a specific marker of the adverse course of the respiratory pathology and the chronic inflammatory development.

**Conclusions:**

Chronic respiratory disease development is associated with the disturbance of the fatty acid composition in erythrocyte membranes and disbalance of the ratio between precursor of pro- and antiinflammatory eicosanoids.

## Background

Diseases of the respiratory system are among the most common diseases and so present a significant medical and social problem as the fourth leading cause of mortality [1,2]. Among chronic respiratory diseases, much attention is paid to chronic obstructive pulmonary disease (COPD) and chronic bronchitis (CB) [3-5]. From 4% to 15% of total adult population suffer from COPD. The frequency of chronic bronchitis in different countries varies in a wide range from 10% to 47% on an average [1]. The views of the researchers on the relationship of CB and COPD have been gradually changing with better understanding of the pathogenesis of these diseases but still they remain obscure. Earlier, the symptoms and morphological changes characteristic of chronic bronchitis were considered as stage 0 or a developing increased risk of COPD [6]. Later, chronic bronchitis was considered as an independent disease which causes no lung function decline [7]. In recent years, the researchers have become convinced that chronic bronchitis may precede the development of COPD [4]. Patients with COPD usually ask for medical help only in the late stages of the disease, when medical treatment cannot prevent the progression of the disease and the development of complications. According to this problem, the possibly early preclinical diagnosis of COPD is urgent [1,2].

Respiratory diseases are accompanied by the development of the chronic inflammatory reaction that greatly aggravates the course and prognosis of the underlying disease [8]. The development of the inflammation in the pulmonary diseases involves also participation of the inflammatory mediators (cytokines, histamine, neutral proteases, and eicosanoids) [9-16]. Particular attention is paid to the lipid mediators - eicosanoids (prostaglandins, leukotrienes, thromboxanes), which are the final products of the metabolism of polyunsaturated fatty acids (PUFAs) [11,13,17]. The violation of the membrane FA composition and FA metabolism is an important factor in the development of the pulmonary diseases [18,19]. Polyunsaturated fatty acids are on one hand the structural components of biological membranes [20,21] and on the other hand they are substrates for synthesis of eicosanoids [22]. Changes in the FA profile of the lipid membranes may lead to violation of the aggregation, and diffusion transfer of membrane components, the activity of membrane-bound enzymes and the expression of receptors, the membrane permeability and transport properties [20,21]. Many functions of immune cells, such as secretion, chemotaxis, and sensitivity to microorganisms depend also on the fluidity of the membrane [23]. The literature does not sufficiently highlight the qualitative and quantitative composition of FAs of cell membranes in chronic pulmonary diseases. Moreover, the results of different authors differ significantly, which may be associated with a variety of biomaterials used for FA analysis (red blood cells, plasma) and the degree of clinical severity of the pulmonary disease [24,25]. There are no available data about possibility of using FA composition as a prognostic test for the complications in the chronic respiratory diseases.

Objective: To assess the fatty acid composition of the membranes of the red blood cells in patients with chronic bronchitis and stable chronic obstructive pulmonary disease.

## Methods

### Subjects

The study involved 40 subjects, 30 of them were patients with diseases of the bronchopulmonary system (19 men, 11 women) aged 23–57 years, including 15 patients with chronic bronchitis (CB) in remission and 15 patients with chronic obstructive pulmonary disease (COPD) of a stable flow (Group A spirometric, GOLD class 1). The studies were performed according to Helsinki Declaration (http://www.wma.net/en/30publications/10policies/b3/index.html). Each subject gave written informed consent for participating in the study, which was approved by the ethics committee of Vladivostok Branch of the Far Eastern Center of Physiology and Pathology of Respiration of SB RAMN - Institute of Medical Climatology and Rehabilitative Treatment. The disease was diagnosed on the basis of the medical history, physical examination, and laboratory tests with post-bronchodilator spirometry. COPD was diagnosed according to the “Global Strategy: diagnosis, treatment and prevention of COPD (GOLD 2011) [1]. Ten healthy subjects were included in the study as a control group; they were ex-smokers or nonsmokers without respiratory infection within at least the last 4 weeks. Exclusion criteria were the presence of professional bronchopulmonary diseases, cardiovascular diseases (coronary heart disease, hypertensive disease) and their complications, diabetes, thyroid disease, acute pathological conditions, and exacerbations of chronic diseases. Spirometry revealed no signs of airflow limitation in patients with CB. COPD was defined by post-bronchodilator test FEV1/FVC <70% (ratio of forced expiratory volume in one second (FEV 1) to forced vital capacity of lungs (FVC).

### Evaluation of the immune system

The immune system was evaluated by the phagocytic activity of the neutrophils, the level of the cytokines in the blood serum. The phagocytic activity of the neutrophils was studied by the Mayansky method [26]. The receptor for TNFα, the levels of tumor necrosis factor (TNF RI (p55), interleukin-8 (IL-8), interleukin-10 (IL-10), the basic fibroblast growth factor (bFGF), and transforming growth factor β1 (TGFβ1) were studied in the blood serum. Cytokine levels were determined by ELISA test using a kit from «Genzyme Diagnostics». Measurements were carried out in 96-well flat-bottomed plates with a Bio-tek Power Wave spectrophotometer (USA).

### Determination of the fatty acids in the erythrocyte membranes

Erythrocyte membranes were prepared by erythrocyte hemolysis with distilled water, centrifugation for 15 min at 14000 rev/min in PBS solution with thrice washing. The red blood cell was taken as a classical model of cell membranes as well as a universal model for the study of pathological reactions: e.g. erythrocyte membranes were used as diagnostic criteria for assessment of different diseases [27]. Lipids were extracted from the membranes of the red blood cells using the solvent system chloroform – methanol, 1:2 (v/v), and then chloroform-methanol (1:1 v/v) and 0.9% sodium chloride were added until a complete phase separation is reached [28]. Fatty acid methyl esters (FAME) were obtained by a sequential treatment of the total lipids with 1% sodium methylate/methanol and 5% HCl/methanol according to Carreau and Dubacq [29] and purified by preparative silica gel thin-layer chromatography, using the silica gel plates developed in benzene. MEFA were analyzed on a Shimadzu GC-2010 (Japan) gas chromatograph equipped with a flame ionization detector, using a fused silica capillary column (Supelcowax-10, 30 m × 0.25 mm i. d. Supelco, Bellefonte, PA). Helium was used as a carrier gas at a linear velocity of 30 cms^–1^. The column temperature was 210 C°, injector and detector temperatures were 250°C. Fatty acids were identified by a comparison with standard mixtures and equivalent chain length values [30]. The results were expressed in relative % of total FAs.

### Statistical analysis

Statistical data processing was performed using the methods of descriptive statistics with Statistica 6.0: the arithmetic means (M) and the standard error of arithmetic means (m). The differences in contents of the fatty acids between the patients were tested using a Student’s *t*-test. Differences were considered statistically significant at *P* < 0.05.

## Results

The research of the immune status in patients with CB and COPD revealed the presence of an inflammatory process (Table 1). The found phagocytic activity of the neutrophils decrease indicates an inhibition of the non-specific immune protection factors. The high TNF-α s-TNFα-RI (p55) value (p<0.001 for all indices) was registered in patients of both groups, CB and COPD, as compared with the control group. It is known that the soluble inhibitor (s-TNFα-RI) promotes long persistence of the primary proinflammatory cytokine (TNF-α) in the blood flow and so leads to development of the unstoppable chronic inflammation [12]. Patients with CB had overproduction of the inflammatory mediators compensated by increased IL-8 (p<0.001) with an immunosuppressive activity [15,16]. The level of IL-8 in patients with COPD did not differ from that in the control group. The level of TGF-β was decreased (p<0.01) in patients with CB and significantly increased (p < 0.001) in patients with COPD. Development of COPD was associated with high values of bFGF (p<0.001). The observed phagocytic activity of the neutrophils fall on the background of the increasing phagocytic activity of neutrophils in patients with CB (TNF-α, s-TNFα-RI (p55)) and COPD (TNF-α, s-TNFα-RI (p55), TGF-β) indicates the development of the chronic inflammatory process [12].

**Table 1:** **Indicators of the immune status in patients with chronic respiratory diseases**, **mean** ± **SE**

**Indicators**	**Healthy subjects n** **=** **10**	**Patient with chronic bronchitis, n** **=** **15**	**Patients with chronic obstructive pulmonary disease n** **=** **15**
PAN, %	64.52 ± 0.68	59.90 ± 0.88*	57.52 ± 1.96*
TNFα, pg/ml	2.60 ± 0.03	7.89 ± 1.47***	^×^4.3 ± 0.3***
sTNF-RI, pg/ml	789.1 ± 7.10	1595.58 ± 141.53***	1523.4 ± 11.0***
bFGF, pg/ml	38.3 ± 0.8	38.93 ± 1.54	^×^53.9 ± 0.3***
TGF-β, pg/ml	1865.4 ± 11.3	1479.17 ± 19.60**	^×^48145.0 ± 20.6***
IL-8, pg/ml	11.5 ± 1.9	17.40 ± 1.20***	^×^13.4 ± 1.6

PAN – phagocytic activity of the neutrophils. Asteriks indicate significant differences of indicators between patients with respiratory diseases and healthy subjects: * for р < 0.05; ** for р < 0.01; *** for р < 0.001. Asteriks indicate significant differences of fatty acid contents between patients with chronic bronchitis and with chronic obstructive pulmonary disease: ^×^ for р < 0.001.

It was found that some saturated fatty acids, such as 12:0 (p <0.01), 14:0 (p <0.01), and 15:0 (p <0.05) were accumulated in erythrocytes of patients with CB, compared to the control group (Table 2). The relative content of 16:1n-7 in the erythrocyte membrane increased (p <0.05) in patients with CB. The relative content of 20:4 n-6 increased by 13% (p <0.05) compared to healthy individuals. The level of 20:5n-3 decreased by 32% (p <0.05) compared to the control group. Reduction of the 20:5n-3 level in the erythrocyte membrane lead to reduction of the content of its metabolite 22:5n-3 by 6% (p <0.05). Changes in the contents of 20:4n-6 and 20:5n-3 lead to a 1.5-fold increase in the ratio 20:4n-6/20:5n-3 (p <0.001).

**Table 2:** **Fatty acid composition of erythrocyte membranes in patients with chronic respiratory diseases**, **mean** ± **SE**

**Fatty acids %**	**Healthy subjects n** **=** **10**	**Patient with chronic bronchitis n** **=** **15**	**Patients with chronic obstructive pulmonary disease n** **=** **15**
12:0	0.18 ± 0.01	0.35 ± 0.07**	
14:0	0.39 ± 0.03	0.65 ± 0.12**	^×××^0.34 ± 0.01
15:0	0.17 ± 0.01	0.24 ± 0.04*	^×××^1.08 ± 0.01***
16:0	23.98 ± 1.28	22.32 ± 1.47	23.16 ± 0.28
16:1n-9	0.21 ± 0.05	0.27 ± 0.03	0.21 ± 0.05
16:1n-7	0.39 ± 0.03	0.51 ± 0.05*	0.53 ± 0.05
17:0	0.35 ± 0.02	0.27 ± 0.03	0.25 ± 0.03
18:0	13.40 ± 0.75	15.27 ± 0.52	16.78 ± 0.40*
18:1n-9	14.84 ± 0.84	14.86 ± 0.45	15.49 ± 0.73
18:1n-7	1.53 ± 0.08	1.33 ± 0.12	^××^1.76 ± 0.05
18:1n-5	0.32 ± 0.02	0.33 ± 0.08	0.36 ± 0.14
18:2n-6	15.75 ± 0.28	15.00 ± 0.16	^×××^12.21 ± 0.61**
18:3n-3	0.15 ± 0.02	0.22 ± 0.09	^×××^0.13 ± 0.03
20:0	0.15 ± 0.035	0.13 ± 0.04	^×××^0.46 ± 0.04***
20:1	0.34 ± 0.07	0.27 ± 0.07	0.23 ± 0.03
20:2n-6	0.25 ± 0.005	0.29 ± 0.03	0.26 ± 0.02
20:3n-6	1.59 ± 0.51	1.45 ± 0.17	1.70 ± 0.20
20:4n-6	12.95 ± 1.65	14.89 ± 0.79*	^×××^18.26 ± 0.50***
20:5n-3	1.23 ± 0.04	0.93 ± 0.09*	^×××^0.56 ± 0.06***
22:4n-6	2.37 ± 0.29	2.38 ± 0.22	^×^3.18 ± 0.31**
22:5n-6	0.37 ± 0.01	0.31 ± 0.04	^×^0.46 ± 0.07
22:5n-3	1.99 ± 0.02	1.88 ± 0.03*	1.82 ± 0.05*
22:6n-3	4.67 ± 0.85	6.09 ± 0.60	5.87 ± 0.39
Sum n-6	32.91 ± 0.10	34.32 ± 0.70*	36.07 ± 0.55**
Sum n-3	8.04 ± 0.12	9.12 ± 0.89	8.32 ± 0.42
20:4n-6/20:5n-3	10.52 ± 0.12	16.01 ± 1.3***	32.60 ± 2.8***

Other fatty acids (10:0, 19:0, 22:0, 14:1, 22:1, 18:2n-5/9, and 20:3n-3) were present in trace amounts (< 0.1%) and are not included into the table.

Asteriks indicate significant differences of fatty acid contents between patients with respiratory diseases and healthy subjects: * for р < 0.05; ** for р < 0.01; *** for р < 0.001; Asteriks indicate significant differences of fatty acid contents between patients with chronic bronchitis and with chronic obstructive pulmonary disease: ^×^ for р < 0.05; ^××^ for р < 0.01; ^×××^ for р < 0.001.

The contents of 15:0 (p <0.001) and 18:0 (p <0.05) 20:0 (p <0.001) in patients with COPD increased as compared to the control group. The relative content of essential linoleic acid 18:2n-6 reduced (p <0.01). In the erythrocyte membranes, the level of 20:4n-6 increased by 41% (p <0.001) as compared to the control group. The relative contents of 20:5n-3 and its metabolite docosapentaenoic acid 22:5n-3 in the erythrocytes decreased, respectively, twice (p <0.001) and by 9% (p <0.05). The study revealed an accumulation of 22:4n-6 (p <0.001) and a rise of the amount of PUFAs n-6 by 10% (p <0.01) as compared to the control group. The ratio of 20:4n-6/20:5n-3 in COPD patients was 3-fold higher (p <0.001) than in the control group.

## Discussion

The results prove the fact that the fatty acid composition of the erythrocyte membranes in patients with chronic respiratory diseases was modified. Moreover, the changes in the physiologically important FAs in patients with COPD and CB were unidirectional. The study revealed a significant accumulation of saturated FAs. Saturated FAs (in particular palmitic and stearic acids) are the building components of the lung surfactant [31]. Therefore, an increased content of FAs with the saturated hydrocarbon chain in the erythrocyte membranes in patients with bronchopulmonary diseases might be an advantage. On the other hand, accumulation of saturated FAs in the cell membrane increases the rigidity of the lipid bilayer and so leads to disruption of the structural and functional characteristics of the cells, to reduction of its fluidity and membrane-dependent enzyme activity, to inhibition of receptor binding by ligands increases the risk of the cell death mediated by apoptosis or necrosis at membrane destruction [25].

Our study has determined a significant breach in the PUFA composition. Patients with CB and COPD have shown an accumulation of 20:4n-6 against a deficiency of 20:5n-3. An imbalance of these PUFAs in patients with COPD is more pronounced than in patients with CB. This fact indicates the aggravation of the FA modification in the cell membranes during the COPD development. The pathogenic significance of the identified modifications is determined by the functional role of lipids in cellular structures. Polyunsaturated FAs perform two functions in cells - structural [20,21] and regulatory [32,33]. The regulatory function is associated with the fact that cells utilize PUFAs as precursors of the synthesis of biologically active substances, eicosanoids. Eicosanoids locally regulate the function of the endothelium, smooth muscle cells, the reaction of the vasodilation, platelet aggregation, microcirculation and inflammation [22,32]. Arachidonic acid (20:4n-6) and its metabolite (22:4n-6) are substrates for the biosynthesis of the eicosanoids, which have inflammatory, chemotactic, and bronchospastic properties [34]. But antiinflamatory eicosanoids with a bronchodilation property are synthesized from eicosapentaenoic acid (20:5n-3) and its metabolic products (22:5n-3 and 22:6n-3) by cyclooxygenase and lipoxygenase reactions [32].

Therefore, a high content of PUFAs of the n-6 family provides inflammatory component effects on the platelet aggregation properties, the immune system functioning, and bronchospasm. The observed increase in the contents of 20:4n-6 and 22:4n-6 in the erythrocyte membrane in patients with chronic bronchopulmonary pathologies indicates an increase of the substrate for formation of the inflammatory (leukotriene B4) and bronchoconstriction (prostaglandin D2) mediators [34]. Besides, the high content of arachidonic acid occurs on the background of a significant deficit of the eicosapentaenoic acid (20:5n-3). These FAs are competitors for the lipooxygenase and cyclooxygenase enzymes [22,34]. It is known that endogenous deficiency of PUFA n-3 in cells alters the physico-chemical properties of the plasma membranes, leads to the receptor malfunction, synthesis of proinflammatory and bronchoconstrictor eicosanoids, and to formation of systemic inflammation [18,32]. The development of the inflammatory response in patients with respiratory diseases is confirmed by the identified imbalance between proinflammatory cytokines and low phagocytic activity of the neutrophils. An increased ratio of 20:4n-6/20:5n-3 which indicates violations in the eicosanoid cycle and the predominance of the synthesis of proinflammatory mediators, became a significant factor of the increased risk of the complication and aggravation of the respiratory pathology. Consequently, the increased 20:4n-6/20:5n-3 ratio can be a specific marker of an unfavorable course of bronchopulmonary diseases. Early detection of violations of the FA composition in patients with respiratory diseases and an adequate correction of these disorders can prevent the development of a more severe pulmonary disease.

## Conclusions

Thus, the study has shown that the development of the chronic respiratory diseases is accompanied by the breach of the fatty acid composition of the erythrocyte membrane. Modification of the fatty acid composition in the membranes of the red blood cells in patients with chronic pulmonary diseases not only leads to disruption of the cell structure skeleton but also to violation of the regulation of the immune response. It is possible that disruption of the lipid components of the cell membrane causes development of the inflammatory process, which is involved in the pathogenesis of chronic bronchitis and chronic obstructive pulmonary disease. Further research is needed to study the metabolism of the fatty acids and eicosanoid formation activity in the respiratory diseases.

## Abbreviations

CB: Chronic bronchitis; COPD: Chronic obstructive pulmonary disease; FA: Fatty acid; bFGF: Basic fibroblast growth factor; IL-8: Interleukin-8; IL-10: Interleukin-10; PUFA: Polyunsaturated fatty acid; TGF β1: Transforming growth factor β1; TNFα: Tumor necrosis factor α; s-TNFα RI (p55): Receptor of tumor necrosis factorαI.

## Competing interests

The authors have no conflict of interest to declare.

## Authors’ contributions

TPN, YKK, VVK, TAG were responsible for the initial conception and design of the study. TPN had primary responsibility for the final content; participated in critical revision of the study. YKK conducted the research and wrote the manuscript. MVA had responsibility for the final content. VVK contributed to the analysis of data, statistical analysis. NVZ performed fatty acid analysis, participated in discussion of the results, writing and translating of the manuscript. TAG participated in critical revision of the study and was responsible for the financial support. All the authors read and approved the final manuscript.
